# Assessment of fNIRS Processing Methods on Active Walking Data: Findings and Implications for Future Research

**DOI:** 10.1093/geroni/igaa057.2868

**Published:** 2020-12-16

**Authors:** Meltem Izzetoglu, Roee Holtzer

**Affiliations:** 1 Villanova University, Villanova, Pennsylvania, United States; 2 Yeshiva University, Bronx, New York, United States

## Abstract

Functional near infrared spectroscopy (fNIRS) studies utilized a limited and inconsistent number of processing algorithms to assess the prefrontal activity during active walking. To address this critical limitation, we have reanalyzed our large dataset of older adults (n=83) who underwent single and dual-task walking (STW and DTW) protocol by applying different hemodynamic conversion parameters and movement and physiological artifact removal methods. Linear mixed effect model results indicated significant increases in oxygenated-hemoglobin (HbO2) with expected decline in deoxygenated-hemoglobin (Hb) from STW to DTW (range of effect sizes: 0.59 to 0.64 for HbO2, 0.18 to 0.32 for Hb) irrespective of the methods used. In addition, intraclass correlations suggested excellent reliability across methods and task conditions (HbO2 range=0.982 to 0.996; Hb range=0.883 to 0.984). These findings support fNIRS as a robust approach for measuring prefrontal activity in older adults during walking and emphasize the importance for establishing explicit guidelines/principles for fNIRS processing.

